# Thermal, Mechanical and Optical Features of Aluminosilicate-Coated Cotton Textiles via the Crosslinking Method

**DOI:** 10.3390/polym10010057

**Published:** 2018-01-09

**Authors:** Alenka Ojstršek, Silvo Hribernik, Darinka Fakin

**Affiliations:** Institute of Engineering Materials and Design, Faculty of Mechanical Engineering, University of Maribor, 2000 Maribor, Slovenia; silvo.hribernik@um.si (S.H.); darinka.fakin@um.si (D.F.)

**Keywords:** zeolites, cotton, pad-dry-thermofix, crosslinking agent, functional properties

## Abstract

The presented study focuses on the development of a pad-dry-thermofix functional coating process using a mixture of microporous aluminosilicate particles in diverse bath formulations to impart UV-ray-blocking, thermal stability and easy-care properties to the cotton fabric. The results of Scanning Electron Microscopy (SEM) and X-ray powder Diffraction (XRD) revealed the presence of three different types of zeolites within the examined sample, i.e., the largest amount being zeolite A, followed by the zeolite X, and the zeolite ZSM-5. The surface characterization results of zeolite-coated/cross-linked textiles provided evidence of acceptable UV-ray-blocking properties and increased thermal stability, as well as enhanced tensile strength and breaking tenacity without considerably decreasing the whiteness degree. Moreover, the dry crease recovery angle increased for the cotton fabric cross-linked via an mDMDHEU, and decreased significantly using 30 g/L zeolites negatively influencing qualitative values. TG/DTA results have proven the enlarged thermal stability of aluminosilicate-coated cotton, although combustion was not prevented.

## 1. Introduction

Increasing requests for functional, comfortable and safe fiber-forming materials, as well as major global competition in this field, prescribe the intensive development of new technologies for the processing of high added-value textiles, and the progress of innovative, but low-cost and environmentally friendly procedures for their finishing. Recently, there has been an augmented demand for textiles and apparel from materials of natural origin that provide greater comfort, and remain fresh and odor free after prolonged usage [1]. Moreover, the use of natural cellulose-based materials has recently become of great interest for numerous new fields of application (protective clothing, clothing for medical and sport activities, interior decorative coverings, technical textiles, etc.), due to the fact that they are readily available in large quantities and easily produced, and because their molecular structures offer excellent potential as a matrix for the design of bioactive, biocompatible, and intelligent materials [2,3]. In spite of various excellent properties, i.e., high flexibility, high mechanical stability, low density, etc., the usage of cellulosic textiles for special applications is limited by certain disadvantages, especially a high level of combustibility, lack of antimicrobial activity and, in some cases, excessive water absorption ability. Thus, the modification of cellulose fibers is required. Numerous techniques are presently being employed for changing the materials’ surface morphologies and, consecutively, their properties, i.e., dip-coating by the dispersion of well-defined nano-oxides [4,5,6], chemical grafting of particles [7], functionalization by inorganic sol-gel coating [8], layer by layer deposition method [9,10], and embedding the (nano)particles on the fibers by crosslinking agents [11]. Therefore, it is significantly important to establish an application technique that can be implemented readily by textile manufacturers [12].

Zeolite particles, as potential material for textile surface modifications, are hydrated crystalline aluminosilicates consisting of three-dimensional frameworks of various numbers of SiO_4_ and AlO_4_ tetrahedra linked together in such a manner as to form channels and cavities within the size ranges 0.3–1.2 nm, filled by cations and water molecules [13]. Due to their particular characteristics, i.e., high thermal and hydrothermal stabilities, high surface area, exchangeable cations, and uniform internal channels and cavities, synthetic zeolites are exceedingly usable for numerous applications that are related to membrane separation, industrial ion exchange, adsorption, solid-acid catalysis processes, etc. [14,15]. The field of surface modification of clothing textiles by zeolites has been researched poorly and has been focused mainly on cotton and polyester surfaces with enhanced thermal stabilities and flame-retardant properties [2,16,17]. The study realized by [18] demonstrated an idea where zeolite 5A was used on wool fabric for the creation of a superhydrophilic surface.

Therefore, the presented research was focused on three main goals, namely: (i) analysis of selected microporous aluminosilicate particle mixtures, (ii) development of an industrially acceptable coating technique for application of particles on the cotton fabric with the inclusion of a crosslinking/easy-care finishing agent, and (iii) characterization of newly gained fabric morphologies, as well as their functional, optical and mechanical features concerning particle concentration and bath formulations.

## 2. Experimental

### 2.1. Materials

In the present study, an industrially bleached (prepared for dyeing) 100% cotton fabric in plain weave was used with a mass of 110 g/m^2^, warp density of 41 threads/cm and weft density of 38 threads/cm, and warp/weft fineness of 20 tex. Before a series of coating trials were started, the source fabric was washed at 40 °C for 30 min using a solution of 2 g/L of standard soap, and afterwards rinsed in warm and cold water and dried at ambient temperature. The zeolites were synthesized and supplied by (Silkem Zeolites Production, Inc., Kidričevo, Slovenia), in the form of very fine powder; composed of 18.08 wt % Na_2_O, 29.24 wt % Al_3_O_3_ and 43.73 wt % SiO_2_. Knittex^®^ 7636 (Huntsman, Dornbirn, Austria)—modified dimethylol dihydroxy ethylene urea with inorganic magnesium salt (mDMDHEU) was employed as a pre-catalyzed, highly reactive crosslinking/easy-care finishing agent with extremely low formaldehyde content, which fulfilled the requirement for standard OekoTex 100.

### 2.2. Preparation of Aluminosilicate Coated Textile

Five or 30 g/L of zeolites (Z) in aqueous finishing formulations with varied pH (pH 11, pH 7 and pH 4) were applied onto cotton fabric during the pad-dry-thermofix procedure and, thereafter, a water solution of 40 g/L of mDMDHEU was utilized as a widely used crosslinking agent on selected zeolite-treated samples. According to the previously optimized methods, the samples were padded twice with a zeolite pad-liquor, by using a two-roll laboratory pad-mangle with 80% wet pick-up, dried for 3 min at a temperature of 100 °C, and (padded twice with pad-liquor that contained the crosslinking agent and) then treated at 150 °C for 5 min as recommended by the supplier of mDMDHEU. With an aim to assess the durability of coatings against washing, all the samples (untreated and zeolite-treated) were washed five times at a temperature of 40 °C for 30 min in a Labomat (W. Mathis AG, Oberhasli, Switzerland) using a solution of 2 g/L of standard soap without optical brighteners (the formulation is given in BS EN 20105-CO1:1993; clause 4.2), and a liquor-to-fabric weight ratio of 50:1. Thereafter, the samples were rinsed in tap water and, finally, dried at room temperature. Samples (before washing and after five washing cycles) were compared using scanning electron microscopy (SEM) (Zeiss Gemini Supra 35 VP, Carl Zeiss NTS GmbH, Oberkochen, Germany) with an aim to visually evaluate the durability of the coatings. Moreover, the add-on (*A*) percentage on the surface of cotton fabric was calculated after each washing cycle according to Equation (1):

(1) $$A=\frac{W_{c}-W_{i}}{W_{i}}\cdot 100\left(\%\right)$$

where *W*_c_ is a weight of dry cotton sample after coating; and *W*_i_ is a weight of dry cotton before coating.

All other analytical measurements of the samples were carried-out after five washing cycles.

### 2.3. Analytical Procedure

#### 2.3.1. X-ray Powder Diffraction (XRD)

XRD measurement of the zeolites was performed at room temperature in an XRD diffractometer PANalytical X’Pert PRO (Malvern Panalytical B.V., Almelo, The Netherlands) using CuKα1 radiation (λ_1_ = 1.5406 Å) over 2*θ* range of 5 do 50° per 100 s and a step size of 0.016°.

#### 2.3.2. Scanning Electron Microscopy (SEM)

For the morphological analysis of zeolites and fabric surfaces, an individual sample was placed on an adhesive carbon band attached to the brass holder of a Zeiss Gemini Supra 35 VP scanning electron microscope (Carl Zeiss NTS GmbH, Oberkochen, Germany), and the SEM images were then taken.

#### 2.3.3. Fourier Transform Infrared Spectroscopy (FTIR)

FTIR measurements of the fabrics were accomplished using an FTIR System Spectrum GX spectrophotometer (Perkin Elmer, Waltham, MA, USA) with a Golden Gate ATR attachment and a diamond crystal. The absorbance spectra were obtained within the range of 4000–650 cm^−1^, with 32 scans and a resolution of 4 cm^−1^.

#### 2.3.4. Optical Properties

The diffuse reflectance spectra (DRS) profiles of the untreated (reference) and zeolite-modified cotton in the 250–700 nm wavebands were recorded on a Lambda 900 UV-Vis-NIR spectrophotometer (Perkin Elmer, Waltham, MA, USA) with an integrated sphere at a scanning speed of 450 nm per min and a resolution of 10 nm. CIE measurements of (un)coated samples were achieved within a spectral range of 400–700 nm wavelengths by a two-ray Spectraflash SF600 Plus spectrophotometer (Datacolor, Luzern, Switzerland) equipped with an Ulbricht sphere and measuring geometry of d/8° under a standard illuminant D65 (LAV/Spec. Incl.) and, thereby, the average CIE whiteness (*W*) and yellowness (*YI*) indices of samples were calculated according to standards ISO 105-J02:1997 and DIN 6167:1980, respectively.

#### 2.3.5. Ultraviolet Protective Properties

The UV shielding capabilities of the fabrics are usually expressed by the ultraviolet protection factor (UPF), which was calculated from the zeolite-modified fabric UV-A and UV-B transmittance. Transmittance values were recorded according to the Australian/New Zealand Standard (AS/NZS 4399-1996) over an ultraviolet spectral region of 280–400 nm wavelengths using a solar-screen Varian Cary 50 spectrophotometer (Agilent Technologies, Santa Clara, CA, USA), fitted with an integrated sphere accessory and a fabric holder accessory. Each measurement was the average of four scans gained by rotating the sample over a 90° angle.

#### 2.3.6. Mechanical Properties and Crease Recovery Angle

Selected mechanical properties (elongation at break, tensile strength and breaking tenacity) of modified fabrics were determined according to Standard ISO 13934-1 using a Textechno statigraph M test machine (Textechno H. Stein GmbH & Co. KG, Moenchengladbach, Germany).

The crease recovery angle (CRA) and qualitative number (*Q*) were determined according to EN 22313. The reported values are the average of five measurements along both the warp and weft directions.

All samples were conditioned before testing for 24 h in a standard atmosphere according to ISO/R 139 at a temperature of 20 ± 2 °C and a relative humidity of 65% ± 2%.

#### 2.3.7. Thermogravimetric Analysis and Vertical Burning Test

The thermogravimetric analysis (TGA) was performed using a thermogravimetric analyzer Q5000IR (TA Instruments, New Castle, Germany), within the temperature range from ambient to 650 °C at a heating rate of 10 °C/min in a continuous air flow using opened Pt holder.

Vertical burning tests were conducted according to standard EN ISO 6940:1999 on vertically oriented specimens (200 mm × 80 mm).

## 3. Results and Discussion

### 3.1. Analysis of Aluminosilicate Particles

**Figure 1:**
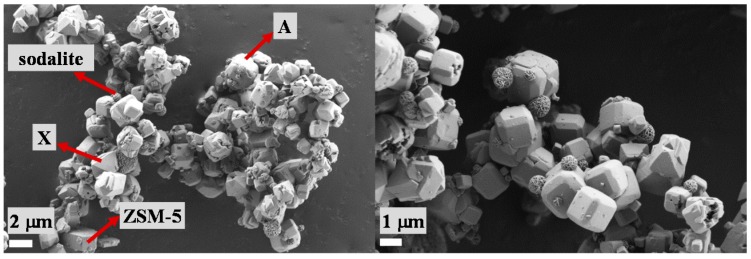
SEM images of used aluminosilicate particles at different magnifications.

**Figure 2:**
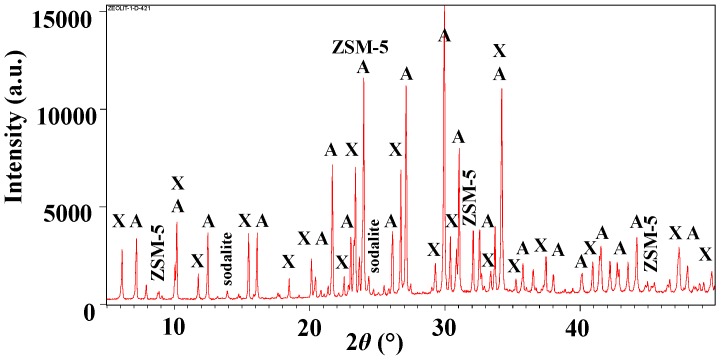
X-ray diffractogram of used zeolite powder.

SEM images of the selected aluminosilicate particles were taken with the intention of studying zeolite surface morphologies for the subsequent modifications of cotton fabric (Figure 1). Furthermore, XRD analysis was accomplished in order to investigate the crystalline nature of zeolites, as shown in Figure 2.

A mixture of three different types of zeolites, in sizes between 2 and 6 μm in different proportions, could be perceived unequivocally from the SEM micrographs in Figure 1. Notably, the examined sample was the most abundant with the cubic crystals of zeolite A, followed by the octahedral crystals of zeolite X, and the pentasil building blocks of zeolite ZSM-5 in moderate amounts, which was also confirmed by the XRD measurement (Figure 2). Furthermore, a high amount of sodalite was noticed, as well as the presence of impurities, probably due to the industrial syntheses and blending procedures.

The XRD pattern in Figure 2 exhibited strong peaks at 2*θ* values characteristic of three different types of zeolites, i.e., A, X, and ZSM-5 [19], with a high crystallization degree as disclosed previously by the SEM micrographs in Figure 1. Some peaks from different zeolites were overlapping and thus, amplifying the intensity of the signals, as compared with the reference XRD patterns for pure zeolite phases [19].

### 3.2. Morphological and Chemical Characterisation of Zeolite-Modified Cotton

**Figure 3:**
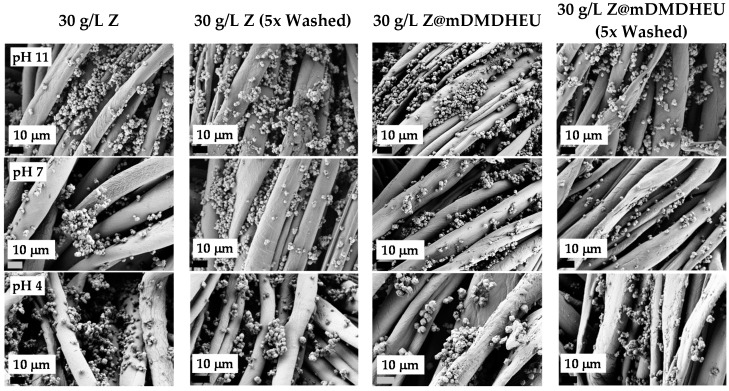
Selected SEM images of cotton: modified by 30 g/L Z (**1st column**); 30 g/L Z/ 5 times washed (**2nd column**); 30 g/L Z@mDMDHEU (**3th column**); and 30 g/L Z@mDMDHEU/ 5 times washed (**4th column**), at pH 11 (**1st row**); pH 7 (**2nd row**); and pH 4 (**3th row**).

The surface morphologies of selected zeolite-modified/washed cotton fabrics were studied by SEM and the specific molecular vibrations on the surfaces by FTIR spectroscopy, with regard to different initial bath compositions, i.e., concentrations of zeolites (Z), pH and presence of the crosslinking agent (mDMDHEU). The gained SEM micrographs are shown in Figure 3.

The SEM micrographs in Figure 3 showed a normal spiral structure of cotton fibers with different surface morphologies, which were created by an application of 30 g/L of zeolites (1st column) or 30 g/L of zeolites in combination with 40 g/L mDMDHEU (3th column) according to the pad-dry-thermofix procedure, by changing the pH of the padding bath (pH 11, pH 7 and pH 4). SEM images in 2nd and 4th columns show that even after five times washing, significant amounts of zeolites are present on cotton and the surface morphologies are very similar to the unwashed samples. In order to confirm those results, the add-on (*A*) has been determined after each washing cycle according to Equations (1). It was calculated that after a second washing cycle, the add-on of zeolites was reduced up to 5.8% (5 g/L Z), 7.2% (30 g/L Z), 3.2% (5 g/L Z@mDMDHEU) and 5.5% (30 g/L Z@mDMDHEU) with negligible differences between bath pHs. After the fourth and fifth washing cycles ca. 75% ± 5% of the zeolites still remain on the cotton fabrics, irrespectively of treatment variables, which indicate satisfactory durability. Therefore, all other analytical measurements were carried-out on five times washed samples.

**Figure 4:**
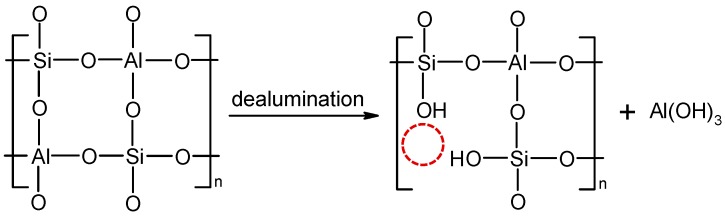
Schematic of dealumination of zeolites in acidic medium.

From Figure 3 it could also be perceived that treatment without crosslinking agent resulted in a higher particle agglomeration on the fiber surface in comparison to the process where zeolite coating was followed by the application of mDMDHEU. As mDMDHEU is a crosslinking agent with extremely low formaldehyde content, it is used widely for the industrial easy-care or durable press finishing of cotton fabrics increasing their dimensional stabilities [20]. According to Ibrahim et al. [21], mDMDHEU forms covalent bonds via the formation of ether links increasing the extent of crosslinking of adjacent cellulose chains and, thus, inhibiting their movement, and in a further reaction, enables the interaction/fixation of different inorganic (nano)particles, i.e., Ag [21], pigment colorant, silicon-based [22], TiO_2_ [23] with active groups of the cellulosic substrates. Based on the published results of the above-cited studies, we are presuming that the three-dimensional frameworks of aluminum silicate particles were combined onto/within the cross-linked cellulose structure through the formation of electrostatic interactions under the given finishing conditions. The most sufficient, but not ideal coating, was achieved when pH 11 baths were employed (the pH was not adjusted). This is also the pH at which the majority of wet-chemical functional treatments of cellulose are performed on an industrial scale without negatively affecting fabric surfaces. On the other hand, when zeolites from pH 4 baths were applied (adjustment by acetic acid), the dealumination of aluminosilicate particles was perceived to a minor extent, which was also reported by [18] when applying 5A zeolite molecular sieves on wool fabrics from a 2 wt % aqueous acetic acid solution. According to the theory, acid attacks the Al–O–Si framework during dealumination via H+ ions breaking down the zeolite structure and, thus, generating large amounts of silanol groups on the zeolite surfaces (Figure 4) which could, in our case, interact with –OH groups of cellulose fibers or with the mDMDHEU crosslinking agent. As presumed by Gonzales et al. [24], different zeolite structure types (the arrangement and the pore size) are known to exhibit different accessibility of the aluminum atoms in the framework and, consecutively, very disparate behavior towards dealumination. Thus, in our case, zeolite ZSM-5 (in the mixture of three zeolites), with a one-dimensional 10-ring pore system, was less prone to dealumination compared to three-dimensional 12- and 8-ring pore systems of zeolite X and A, respectively.

In spite of the partly positive dealumination phenomenon, some deleterious effects on the morphology of the native cellulose fiber were observed using pH 4 baths, as expected to a minor extent, on account of acid hydrolysis [25].

**Figure 5:**
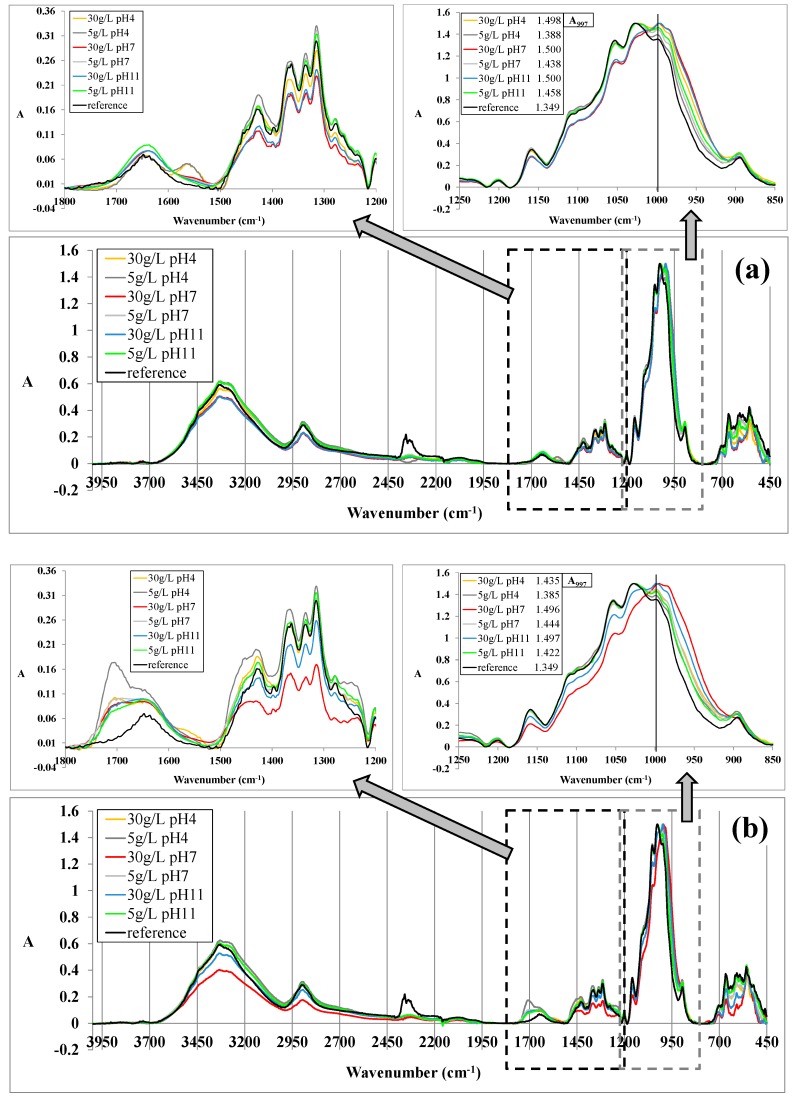
Normalized FTIR spectra (at 1850 cm^−1^) of cotton samples modified by: (**a**) 5 or 30 g/L Z in bath with different pH; and (**b**) two-bath padding with 5 or 30 g/L Z@mDMDHEU.

The aforementioned results are in accordance with the results obtained additionally by FTIR spectroscopic measurements (Figure 5). With the aim to compare the absorbance intensities of characteristic peaks of diversely treated cotton fabric, the FTIR spectra were normalized at a chosen wavenumber of 1850 cm^−1^, which remained unaffected during surface modification.

Figure 5a,b depict FTIR patterns of the cotton surfaces before (reference) and after treatment with typical peak positions for cellulose, including stretching absorption of free hydroxyl groups at 3450–3200 cm^−1^, C–H stretching vibrations at 2890 cm^−1^, water molecules at ~1645 cm^−1^, C–H bending vibrations within the glucose ring at 1426, 1369 and 1315 cm^−1^, asymmetric stretching of C–O–C at 1152 cm^−1^, and C–O stretching vibrations at 1053 and 1028 cm^−1^, as also interpreted fully by [1,26]. Although all the gained spectra were quite similar due to the overlapping of the characteristic absorption peaks of cellulose and aluminosilicate particles, some absorption bands were evidently changed/appeared for Z or Z@mDMDHEU-treated samples, i.e., the peaks at 1028 cm^−1^ were shifted toward a higher wavenumber and peaks characteristic for C–H and O–H vibrations were reduced, implying the efficient surface modification of cellulose fibers. Furthermore, two new peaks appeared besides the above-mentioned, as could be perceived from Figure 5b, at 1708 cm^−1^ and 1239 cm^−1^ on account of the carbonyl stretching and N–H vibrations of –CO–NH–R bonds [27], which were formed as a result of successful high-temperature crosslinking between adjacent cellulose chains and the mDMDHEU.

In order to monitor how effective the zeolite modification was, the relative absorbance intensities of the Si–O– band at 997 cm^−1^ wavenumber (A_997_) were emphasized (vertical lines on small Figure 5a,b) as a function of bath composition, its pH and zeolite concentration. It was found that the relative absorbance values of modified samples were larger compared to the untreated cotton sample and, moreover, A_997_ values were amplified by enlargement of zeolite concentration, as well as bath pH.

### 3.3. Diffuse Reflectance Spectra Profile Determination and CIE Measurement

**Figure 6:**
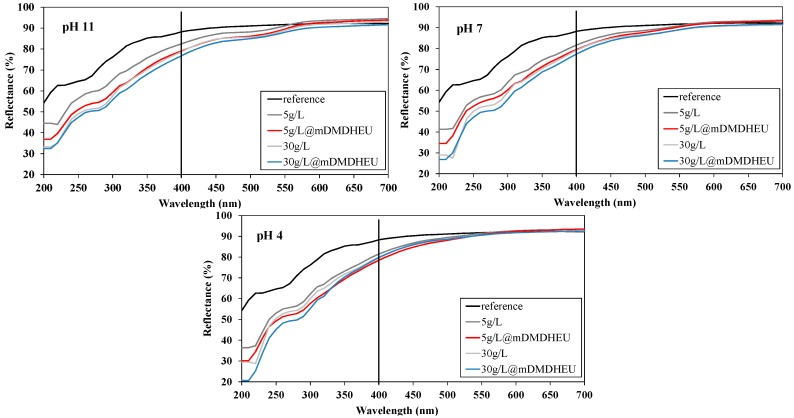
Diffuse reflectance spectra of untreated and zeolite-coated cotton using different bath pH: 11, 7 and 4.

**Figure 7:**
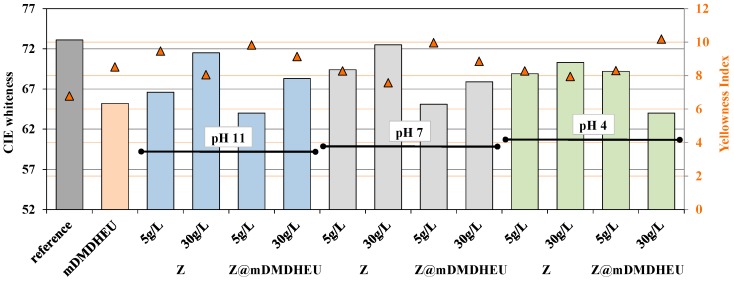
CIE whiteness and yellowness index of untreated cotton, and cotton coated by two concentrations of zeolites (Z) in aqueous dispersions with different pH; and after-treatment with mDMDHEU.

The DRS profiles of the cotton fabrics in both the UV (wavelengths between 250 and 400 nm) and Vis (wavelengths between 400 and 700 nm) regions before and after zeolite modification are depicted and compared in Figure 6. Moreover, based on the presumption that zeolites are white pigments producing a white color when applied onto the material’s surface and, on the other hand, that the crosslinking agent could cause fabric yellowness under high treatment temperatures, the influence of coatings on the samples’ visual changes was given by CIE measurement, and by calculated whiteness (*W*) and yellowness (*YI*) indices (Figure 7).

The upper curve in Figure 6 shows the reflectance values of industrially bleached untreated fabric (reference) with no significant absorption peak (reflectance minimum), meaning that optical brighteners, which could interfere with the coating procedure, were not present on the fabric surface. After the application of zeolites, the optical properties were changed in both UV and visible regions regarding the concentration of zeolites and bath pH, as well as the presence of mDMDHEU on the fabrics. Also, an extra absorption peak occurred in the UV-B region at 260 nm for all coated samples due to light scattering at lower wavelengths, especially by larger zeolite agglomerates. As can be seen from the curves, zeolites significantly enhanced the absorption ability of UV-rays (reflectance values are lower in comparison to the reference sample). The stronger the UV absorption intensity of the zeolite-modified textiles, the higher the UV protection ability was; which is in good agreement with the UPF results disclosed additionally in Figure 7, as well as with the findings of the study accomplished by [16].

The results in Figure 7 revealed visually perceivable changes in whiteness/yellowness between the reference and zeolite-coated samples, as well as between bath pH. Samples treated with zeolites were less white than the reference sample, although the higher zeolite concentration enlarged whiteness degree in general. As expected, the application of mDMDHEU at a 150 °C thermofixing temperature elevated fabric yellowness irrespective of bath composition.

### 3.4. UV-Protective Properties

**Figure 8:**
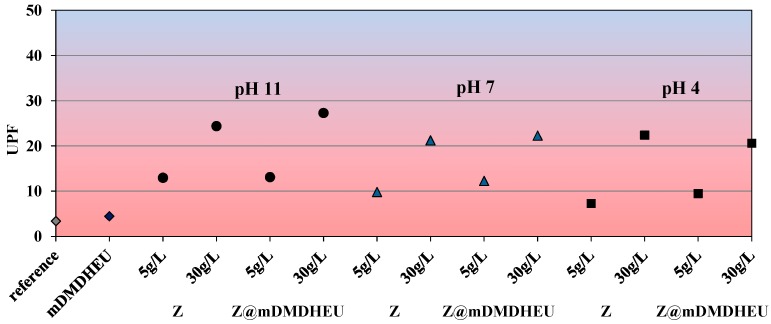
UPF of zeolite-modified cellulose samples.

The influence of the bath compositions on the cotton fabric UV-blocking functionalities was followed by means of spectrophotometry. The gained ultraviolet protective factor (UPF) results are presented in Figure 8.

It was evident from Figure 8 that the untreated cotton had UPF 3.4, indicating a non-ratable UV protection level. After the applications of 5 and 30 g/L of zeolite particles during the pad-dry-thermofix procedure, the fabric UPFs increased exceedingly, as expected from the literature [16], i.e., up to values of 24.4 (30 g/L Z at pH 11), and 27.3 (30 g/L Z@mDMDHEU at pH 11), meaning good to very good UV protective properties with regard to ASTM guidelines for labelling UV-protective clothing (ASTM D 6603), which was in good agreement with the above-presented DRS profiles in the UV region (Figure 6). Although the achieved UPFs of all the zeolite-modified/washed samples were lower than the value of 55 declared as excellent protection (UPF 50+), the gained results confirmed that zeolites impart functionality to cellulose fabrics against the transmittance of harmful solar UV rays. Further employment of dyestuffs/printing pigments and finishing agents could also improve the UPFs, as is well-documented [1,16].

### 3.5. Mechanical Performance

SEM and FTIR observations, together with the UPF results, led us to select those samples that were treated with pH 11 baths for mechanical property determination. Thus, elongation at break, tensile strength and breaking tenacity were compared to the untreated fabric in both warp and weft directions in order to see if the zeolite particles and easy-care finishing agent had any adverse effect on mechanical performance. Additionally, the crease recovery angles (CRA) and qualitative numbers (*Q*) were calculated. The magnitude of the CRA is an indication of the fabric’s ability to recover from accidental creasing, and the *Q* value is the ability of fabric recovery from creasing in one or both directions with regard to an ideal situation of 100% recovery. The obtained results are summarized in Table 1.

From Table 1, it could be perceived that, after zeolite particle application, the elongation at break was decreased moderately, i.e., from 8.65% to 8.51% (warp) and from 14.31% to 11.91% (weft) and, on the other hand, tensile strength and breaking tenacity were intensified significantly in both directions, as was also reported by [18]. On the other hand, when modified dimethylol dihydroxy ethylene urea was applied as a crosslinking/easy-care finishing agent, all mechanical properties decreased negligibly in comparison with the properties of zeolite-modified fabric due to the loss of flexibility as mentioned in [20].

In addition, the dry CRA increased for the cotton fabric cross-linked with 40 g/L mDMDHEU up to 93.2° (warp) and 82.4° (weft) in comparison to the CRA of the untreated reference sample, i.e., 63.1° in both directions—an improvement of ca. 48% and 31%, respectively—which is in agreement with the study carried-out by [28]. After crosslinking of cellulose with mDMDHEU, the formation of ether linkages inhibited the movement of molecular chains in the less-oriented or amorphous region of cellulose, causing increments in the CRA values [20,29]. On the other hand, the application of 30 g/L of zeolites deteriorated the CRA significantly and, consequently, negatively influenced the *Q* values, irrespective of the mDMDHEU applied. Crease recovery performance can be enhanced by using a higher amount of mDMDHEU individually or in combination with different softeners as is also well-described by [28].

### 3.6. Thermal Degradation and Burning Behaviour

**Figure 9:**
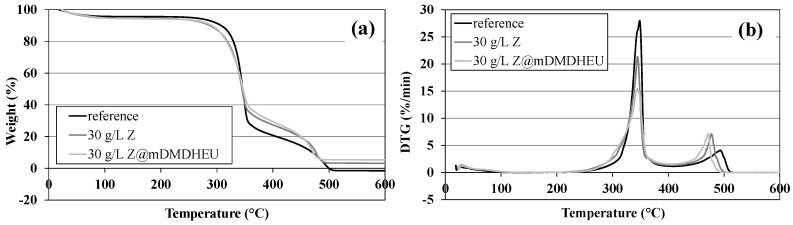
(**a**) TG; and (**b**) DTG curves of untreated and zeolite-coated cotton fabrics.

In order to determine the effect of aluminosilicate coating on the flame retardancy of a cotton fabric, TG and DTA analyses were employed (Figure 9) on selected samples treated with higher concentration of Z in aqueous dispersions with pH 11. The specific degradation temperatures/weight loss and final char yields at 600 °C derived from Figure 9 are summarized in Table 2.

**Figure 10:**
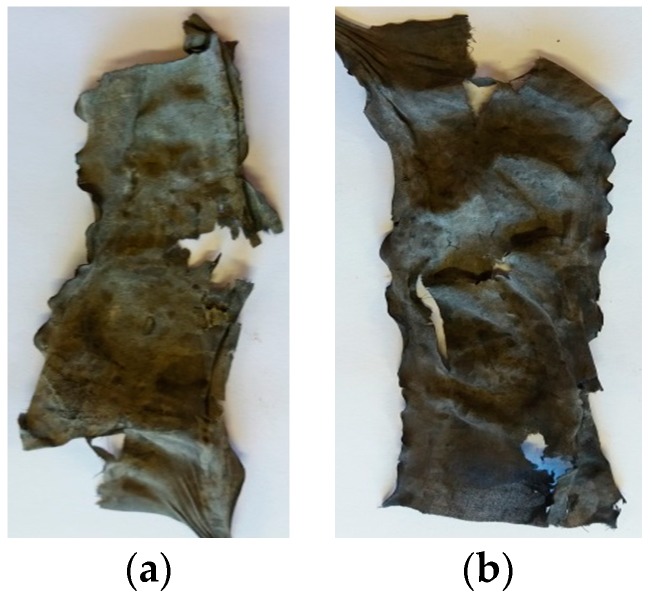
Cotton samples after vertical burning test modified by: (**a**) 30 g/L Z; and (**b**) 30 g/L Z@mDMDHEU.

Figure 9a shows the weight (%) and Figure 9b derivative weight (%/min) of the untreated cotton fabric (reference) and fabric treated with 30 g/L Z and 30 g/L Z@mDMDHEU in air atmosphere, respectively, as a function of temperature. The TG curves (Figure 9a) of all samples reveal that the thermo-oxidative decomposition process of cotton fabrics includes two stages, i.e., 220–400 °C and 400–530 °C. The weight loss of below 220 °C is due to the release of physically adsorbed water (4.2% for untreated, 5.3% for Z and 5.4% for Z@mDMDHEU—Table 2). The first stage was elapsed in the region of 220 up to 400 °C with exothermic peaks at 349 °C (reference) and 345 °C (both zeolite-treated), respectively, where the main pyrolysis and the weight loss occurred, which is consistent with the dehydration and decarboxylation reactions generating combustible gases like aldehydes, ketones, ethers, etc. [29]. The second degradation stage occurred in the region from 400 °C up to 530 °C, which corresponds to the decomposition of char formed in the second stage [30]. Above that range, a plateau is reached, indicating that degradation has been completed. From DTG curves (Figure 9b), it could be perceived that the main decomposition peaks of samples treated with zeolites are lower in comparison with the main peak of untreated cotton fabric. This can be attributed to the fact that zeolite-treated samples generate fewer combustible gases during decomposition, and have a slower decomposition rate as compared to the untreated fabric. Herein, the sample treated with 30 g/L Z@mDMDHEU has the highest char yield (5.3%), followed by the sample treated with 30 g/L Z (3.1%) and the untreated sample (no residue), which is in a good agreement with the results of vertical burning test (Figure 10).

Figure 10 shows the influence of applied zeolite particles on the flammability of cotton fabric. The untreated samples burned through their whole length with a small amount of ashes remaining (figure not shown). Samples treated with 30 g/L Z (Figure 10a) and 30 g/L Z@mDMDHEU (Figure 10b) also burned through their whole length, but slower as compared to the reference sample, i.e., ignition time of 3 s (reference), 11 s (Z) and 13 s (Z@mDMDHEU). A significant increase in the amount of the final residue of modified samples indicated that the aluminosilicate particles were able to retard further degradation of the char formed during the burning.

## 4. Conclusions

The industrially synthesized aluminosilicate particles in aqueous finishing formulations by varied pHs (pH 4, 7 and 11), and a commercially available mDMDHEU as a crosslinking agent were selected for the functionalization of cotton fabric using a conventional pad-dry-thermofix procedure. From the SEM, FTIR and XRD analyses of aluminosilicate particles, it could be concluded that the examined samples were composed of the cubic crystals of zeolite A, the octahedral crystals of zeolite X, and the pentasil building blocks of zeolite ZSM-5 in different proportions, and sizes of ca. 2–6 μm.

Treatment without a crosslinking agent resulted in a higher particle agglomeration on the fiber surface in comparison to the process where zeolite coating was followed by the application of mDMDHEU, as confirmed by SEM and FTIR. The most sufficient, but not ideal coating, was achieved when alkaline (pH 11) baths were employed. On the other hand, some deleterious effects on the cellulose morphologies were noticed employing acidic baths (pH 4), as well as dealumination of zeolites. SEM micrographs and add-on percentage prove the durability of the surface-modifying coatings also after five washing cycles. Furthermore, this research reports acceptable UV-ray-blocking properties of zeolite-coated/mDMDHEU crosslinked cotton fabrics (up to UPF 27.3), as governed by the amounts of zeolites applied. The fabric whiteness degree decreased considerably when mDMDHEU was applied, irrespective of the bath pHs. Moreover, the mechanical performance was evaluated on selected zeolite-coated samples. The results proved that aluminosilicate particles reduced the elongation at break moderately and, on the other hand, enlarged the tensile strength and breaking tenacity in both, weft and warp directions considerably. Crosslinking with mDMDHEU, which also acts as an easy-care finishing agent increased the fabric dry crease recovery angles, positively influencing the qualitative values. Finally, the applied zeolite particles increase the thermo-oxidative stability of cotton, although they do not prevent burning.

## Figures and Tables

**Table 1:** Mechanical properties, crease recovery angle (CRA) and qualitative number (*Q*).

Sample	Elongation at Break (%)	Tensile Strength (N)	Breaking Tenacity (cN/tex)	CRA (°)	*Q* (%)
Reference warp weft	8.65 ± 0.37 14.31 ± 0.38	421 ± 22 54 ± 18	441 ± 22 371 ± 19	63.1 63.1	17.1 17.1
mDMDHEU warp weft	8.12 ± 0.27 13.26 ± 0.28	409 ± 27 322 ± 28	397 ± 29 341 ± 31	93.2 82.4	41.3 36.4
5 g/L Z warp weft	9.24 ± 0.71 13.80 ± 2.20	495 ± 33 389 ± 15	518 ± 34 407 ± 16	56.7 54.6	15.9 14.3
5 g/L Z@mDMDHEU warp weft	7.49 ± 0.59 10.19 ± 1.60	435 ± 18 363 ± 28	456 ± 18 380 ± 29	91.3 89.4	36.3 34.1
30 g/L Z warp weft	8.51 ± 0.26 11.91 ± 1.08	504 ± 36 414 ± 17	528 ± 37 434 ± 18	51.3 52.5	13.3 15.1
30 g/L Z@mDMDHEU warp weft	8.31 ± 0.68 10.30 ± 0.81	501 ± 28 372 ± 33	524 ± 29 389 ± 34	66.8 61.7	16.4 13.8

**Table 2:** The TG/DTG measurement data.

Sample	*T* of Exothermic Peaks (°C)		Weight Loss (%)			Residual at 600 °C (%)
	1st	2nd	<220 °C	220–400 °C	400–530 °C	
Reference	349	494	4.2	75.1	22.2	-
30 g/L Z	345	477	5.3	67.2	24.1	3.1
30 g/L Z@mDMDHEU	345	471	5.4	65.2	23.9	5.3
